# Asymptotic Information-Theoretic Detection of Dynamical Organization in Complex Systems

**DOI:** 10.3390/e23040398

**Published:** 2021-03-27

**Authors:** Gianluca D’Addese, Laura Sani, Luca La Rocca, Roberto Serra, Marco Villani

**Affiliations:** 1 Department of Physics, Informatics and Mathematics, University of Modena and Reggio Emilia, 41125 Modena, Italy; gianluca.daddese@unimore.it (G.D.); luca.larocca@unimore.it (L.L.R.); rserra@unimore.it (R.S.); 2 Department of Engineering and Architecture, University of Parma, 43124 Parma, Italy; laura.sani@unipr.it; 3 European Centre for Living Technology, 30123 Venice, Italy; 4 Institute for Advanced Studies, University of Amsterdam, 1012 GC Amsterdam, The Netherlands

**Keywords:** chi-squared approximation, cluster index, integration, mutual information, relevance index, relevant subset

## Abstract

The identification of emergent structures in complex dynamical systems is a formidable challenge. We propose a computationally efficient methodology to address such a challenge, based on modeling the state of the system as a set of random variables. Specifically, we present a sieving algorithm to navigate the huge space of all subsets of variables and compare them in terms of a simple index that can be computed without resorting to simulations. We obtain such a simple index by studying the asymptotic distribution of an information-theoretic measure of coordination among variables, when there is no coordination at all, which allows us to fairly compare subsets of variables having different cardinalities. We show that increasing the number of observations allows the identification of larger and larger subsets. As an example of relevant application, we make use of a paradigmatic case regarding the identification of groups in autocatalytic sets of reactions, a chemical situation related to the origin of life problem.

## 1. Introduction

The identification of emergent structures in complex dynamical systems is a very difficult task with broad applications. In particular, the formation of intermediate-level dynamical structures is of high interest for what concerns biological as well as artificial systems. This phenomenon is among the most intriguing ones in natural as well as in artificial systems, and a fascinating aspect is its “sandwiched” nature [1]. While past emergence examples were focused on bottom-up emergence in two-level systems, like, for instance, Benard–Marangoni convection cells emerging from the interaction of water molecules under the influence of temperature gradients [2], more recent work points out that in many interesting cases the new entities and levels emerge between preexisting ones [1,3,4]. The paradigmatic example may be that of organs and tissues in multicellular organisms: both the lower (cellular) level and the upper (organism) level predate the appearance of the intermediate structures. Other examples come from the physical world (e.g., mesolevel structures in climate), social systems (e.g., factions within political parties and the parties themselves), or socio-technical systems (e.g., communities in social networks). Very often artificial (and sometimes social) architectures have been devised precisely to stimulate the formation of these mesolevel structures, but here we are concerned with structures that come into being by spontaneous processes, even though their formation may be eased or hindered by an external design.

A central question is then that of identifying the emerging “things”: these may be either static entities or dynamical patterns, or some mixture of the two. The identification of these configurations is seldom simple, because of the more-than-binary relationships among variables, the multiple memberships of system entities or the fuzziness of boundaries among groups. In Network Science [5,6], static emergent structures take the form of topological features, like, e.g., motifs in genetic networks or communities in a broader context; in particular, in the case of socio-technical systems there is an extensive literature on community detection [7,8]. However, most methods are based on static features (such as link distributions or topologies), whereas the system’s elements may work in a coordinated manner even though they are not directly linked, because of the effects of the dynamical laws. If the topology were regular, these nodes might be identified by visual inspection, but in the case of irregular topologies this approach seems hopeless.

Building on the information-theoretic approach proposed by Tononi [9,10], this paper presents a methodology for the identification of mesolevel objects in a system. As these objects may have a topological as well as a dynamical nature, and they typically play a central role in the system’s dynamics, we will refer to them as *relevant subsets*, and we will refer to the indices used for their identification as *relevance indices*. The identification of relevant subsets is a hard task, as discussed above, so we will show here the general schema of a promising approach, compare some indices, and show some results. In particular, in this work we apply our methodology to a relevant case, in which we look for the dynamic organizations (here recognized as groups of variables in dynamic relationship) responsible for the observed behaviors. Specifically, we analyze the results of an experiment frequently performed to examine a system: the observation of its responses when it is subject to solicitations—in our case, distinct groups of autocatalytic chemical reactions in which silencing actions are performed one chemical species at a time. To each solicitation (a silencing) follows a response of the system (a set of chemical species change their activity). We will show that from the observations of the spread of changes it is possible to infer the presence and the composition of dynamically coordinated groups of chemicals, while remarking that there are still open questions to answer.

Our approach will be presented in Section 3, after the necessary background material has been introduced in Section 2, and it will be assessed in Section 4, both from simulation and empirical perspectives; some conclusions will be drawn in Section 5.

## 2. Methodological Background

Section 2.1 sets our mathematical notation and introduces the information-theoretic concepts used in this paper; see Cover and Thomas [11] for a proper introduction to information theory. Section 2.2 presents the Cluster Index (CI) proposed by Tononi et al. [9] in the context of neuroimaging.

### 2.1. Information-Theoretic Preliminaries

Let $X_{S}={\left\{X_{u}\right\}}_{u\in S}$ be a random vector indexed by a finite set of elements $S=\{u_{1},u_{2},\cdots ,u_{k}\}$ in some system of interest. We assume ∅ ⊂ *S* ⊂ *U*, where U={1,⋯,q} is the set of all elements in the system and ∅ denotes the empty set. As a special case, if $S=\{u_{1}\}$ is a singleton, $X_{\left\{u_{1}\right\}}=X_{u_{1}}$ is a random variable corresponding to an individual element in the system. The idea is that the vector $X_{U}={\left\{X_{u}\right\}}_{u\in U}$ models a noisy reading of the system’s state and by considering $X_{S}$ we focus on the subsystem formed by the elements in *S*.

The *entropy* of $X_{S}$, assuming a finite alphabet (set of possible values) for all variables, is defined as

(1) $$H\left(S\right)=-\sum_{x_{1}\in X_{1}}\cdots \sum_{x_{k}\in X_{k}}p_{S}\left(x_{1},\cdots ,x_{k}\right)\log p_{S}\left(x_{1},\cdots ,x_{k}\right),$$

where $X_{j}$ is the alphabet of variable $X_{u_{j}}$, for j=1,⋯,k, and 

(2) $$p_{S}\left(x_{1},\cdots ,x_{k}\right)=P\left\{X_{u_{1}}=x_{1},\cdots ,X_{u_{k}}=x_{k}\right\},\;x_{1}\in X_{1},\cdots ,x_{k}\in X_{k},$$

is the probability mass function of $X_{S}$. The base of the logarithm used in (1) determines the unit of information: a *bit*, if binary logarithms are used, or a *nat*, if natural logarithms are used. If $H_{2}$ denotes the entropy in bits, the change of logarithm base formula gives $H_{e}=\ln\left(2\right)H_{2}$ for the entropy in nats; more generally, if $H_{b}$ is the entropy with logarithm base *b*, then $H_{b}=\log_{b}\left(e\right)H_{e}$. In the following, we use nats (unless noted otherwise).

Following Tononi et al. [10], when $S=\{u_{1},\cdots ,u_{k}\}$ with k≥2, we define the *integration* of $X_{S}$ as

(3) $$I\left(S\right)=-H\left(S\right)+\sum_{j=1}^{k}H\left(u_{j}\right),$$

where $H(u_{j})$ is the entropy of the individual variable $X_{u_{j}}$. It can be shown, by means of the chain rule of probability, that I(S) is always positive and vanishes if $X_{u_{1}},\cdots X_{u_{k}}$ are stochastically independent. Watanabe [12] introduced (3) as a measure of “total correlation” among the variables indexed by *S*. In the special case k=2, the integration of $X_{\left\{u_{1},u_{2}\right\}}$ reduces to the *mutual information* between $X_{u_{1}}$ and $X_{u_{2}}$; see Cover and Thomas ([11], Ch. 2).

Now let $X_{S}^{\left(1\right)},\cdots ,X_{S}^{\left(n\right)}$ be a random sample of observations from the unknown distribution of $X_{S}$. We estimate the distribution of $X_{S}$ by means of the *empirical distribution* of $X_{S}^{\left(1\right)},\cdots ,X_{S}^{\left(n\right)}$, whose probability mass function ${\hat{p}}_{S}\left(x_{1},\cdots ,x_{k}\right)$, $x_{1}\in X_{1},\cdots ,x_{k}\in X_{k}$, is given by the relative frequencies of the possible values of $X_{S}$ in the sample. Using this distribution in (1), we obtain the *empirical entropy* of $X_{S}^{\left(1\right)},\cdots ,X_{S}^{\left(n\right)}$, which we denote by $H_{n}\left(S\right)$. Similarly, using empirical entropies in (3), we define the *empirical integration* $I_{n}\left(S\right)$ of $X_{S}^{\left(1\right)},\cdots ,X_{S}^{\left(n\right)}$. More elaborate entropy estimators are available [13,14,15], but in this work we focus on making the most out of the simplest one.

It has been known since Miller and Madow [16] that, on average, the empirical entropy $H_{n}\left(S\right)$ underestimates its theoretical counterpart H(S):

(4) $$E\left[H_{n}\left(S\right)\right]=H\left(S\right)-\frac{c(S)-1}{2n}+O\left(\frac{1}{n^{2}}\right),$$

where $c\left(S\right)=\prod_{j=1}^{k}\left|X_{j}\right|>1$ is the number of cells in the table indexed by *S* and it is understood that $p_{S}\left(x_{1},\cdots ,x_{k}\right)>0$ for all $x_{1}\in X_{1},\cdots x_{k}\in X_{k}$; note that $|X_{j}|$ denotes the cardinality of $X_{j}$. It follows that the empirical integration overestimates its theoretical counterpart:

(5) $$E\left[I_{n}\left(S\right)\right]=I\left(S\right)+\frac{d_{k}}{2n}+O\left(\frac{1}{n^{2}}\right),$$

where

(6) $$d_{k}=c\left(S\right)-1+k-\sum_{j=1}^{k}\left|X_{j}\right|$$

is a strictly positive integer. In the special case k=2, first studied by Miller [17], the quantity in (6) can be written as $d_{2}=\left(\right|X_{1}\left|-1)(\right|X_{2}\left|-1\right)$; see Luce [18].

### 2.2. Cluster Index

The Cluster Index (CI) is an information-theoretical measure proposed in the 1990s by Tononi and Edelman [9,10], within researches on human brain processes, whose purpose is the identification of subsets of variables in a dynamical system that behave in a coordinated way, while having a relatively limited exchange of information with the rest of the system; these subsets can then be used to describe the whole system organization. Given a subset $S=\{u_{1},\cdots ,u_{k}\}$ of elements in a system indexed by U={1,⋯,q}, where 1<k<q, Tononi et al. [10] defined the integration of *S* as in (3) and considered it as a proxy for the degree of coordination among the *k* variables indexed by *S*. Then, Tononi et al. [9] considered the mutual information between *S* and U\S

(7) M(S)=H(S)+H(U\S)−H(U)

as a measure of the mutual dependence between the subset *S* and the rest of the system U\S, and defined the CI as the ratio between the integration of *S* and the mutual information between *S* and U\S:

(8) $$\mathrm{CI}\left(S\right)=\frac{I(S)}{M(S)}.$$

In practice, analyses resort to the empirical version ${\mathrm{CI}}_{n}\left(S\right)=I_{n}\left(S\right)/M_{n}\left(S\right)$, based on the relative frequencies observed in a sample of system states.

High values of (8) correspond to subsets of *U* where the internal coordination exceeds the exchange of information with the rest of the system, allowing in such a way the identification of interesting groups of variables. As the value of the CI depends on the size of the group under examination, as well as on the size of the system, this index should be normalized before groups of different size can be compared. Tononi et al. [10] proposed as normalizing constants for the numerator and denominator of (8) the averages of integration and mutual information over groups of equal size embedded in a system, called the *homogeneous system*, with the same number of variables and no dynamical organization:

(9) $${\mathrm{CI}}_{n}^{★}\left(S\right)=\frac{I_{n}\left(S\right)/{\langle I_{n}\left(k\right)\rangle }_{0}}{M_{n}\left(S\right)/{\langle M_{n}\left(k\right)\rangle }_{0}}.$$

Eventually, the *z*-score of the normalized CI (numerically identical to the *z*-score of the CI) can be used as an evidence index:

(10) $$z{\mathrm{CI}}_{n}\left(S\right)=\frac{{\mathrm{CI}}_{n}^{★}\left(S\right)-{\langle {\mathrm{CI}}_{n}^{★}\left(k\right)\rangle }_{0}}{{\mathrm{sd}}_{0}\left({\mathrm{CI}}_{n}^{★}\left(k\right)\right)}=\frac{{\mathrm{CI}}_{n}\left(S\right)-{\langle {\mathrm{CI}}_{n}\left(k\right)\rangle }_{0}}{{\mathrm{sd}}_{0}\left({\mathrm{CI}}_{n}\left(k\right)\right)};$$

see Villani et al. [19]. Finally, and interestingly, the *z*CI index is related to the identification of dynamical criticality in complex systems, see Roli et al. [20,21].

## 3. Proposed Methodology

In Section 3.1, we discuss some limitations of the Cluster Index, and we propose a methodology to overcome these limitations. The methodology requires the use of indices to evaluate groups of interest: the identification and comment of these indices is the focus of the current work. In Section 3.2, we show that in homogeneous systems the empirical integration follows an asymptotic chi-squared distribution: this knowledge can be used in different ways, generating different indices. In Section 4, these indices will be applied in different situations, which will allow us to identify the *z*-score of the integration as the most effective one.

### 3.1. Searching for Relevant Subsets

#### 3.1.1. Challenges

Implementing the CI index presents two main challenges:

(a) The cardinality of the set of all possible subsets of a set is gigantic. However, even beforehand the computational effort needed to deal with this amount of data, it is noteworthy that this wide set contains many groups included in others and a huge number of partially overlapping groups. All these situations require further analyses to assess their actual relevance or independence. Indeed, a high index value is not sufficient to characterize a relevant subset, because such a value might result from the presence of a smaller subset characterized by a higher coordination among variables. Conversely, a set having a high index value might reach an even higher value, if some other relevant variables are added to it.
(b) It is burdensome to compute the averages of integration and mutual information on a suitable homogeneous system. Even though simulations from the homogeneous system are straightforward, they have to be repeated for all subsets of interest, which results in very long computing times. Furthermore, a specific homogeneous system has to be selected for the simulations, which introduces an unwelcome degree of arbitrariness in the analysis.

We address challenges (a) and (b) by using the integration alone (through its *z*-score, *z*I in the following) within an iteration scheme like the one presented by Villani et al. [22]. This results in an efficient framework for the identification and subsequent enlargement of dynamically interesting groups.

Use of integration alone enables us to deal with challenge (b) by means of an asymptotic approximation that holds for all systems with independent variables and does not require to simulate from any of them: as shown in Section 3.2, for large *n*, the integration, multiplied by twice the number of observations, approximately follows a chi-squared distribution with degrees of freedom depending on the number of variables belonging to the analyzed subgroup and on the cardinality of their alphabets; such an approximation can be used to obtain *z*-scores with negligible computational effort.

Using the integration alone also deals with a problematic aspect of the division by mutual information: low values of M(S) can derive from a low information exchange between the subgroup *S* and every element of the rest of the system, or from a high exchange of information between *S* and a small part of the rest of the system, while the other parts are not involved in the exchange. The (*z*-score of the) CI index does not distinguish between the two situations [23].

#### 3.1.2. The Iterative Sieving Method

Challenge (a) requires a procedure to compare different subgroups. We suggested [24,25] a comparison procedure (sieve, or sieving algorithm, in the following) based on the consideration that if a set A is a proper subset of a set B and ranks higher than B, then A should be considered more relevant than B. Therefore, the sieve keeps only those sets that are not included in, nor include, any other set with higher *z*I. The comparison procedure is therefore composed by two basic steps: (i) detection of relevant variable sets based on the computation of the *z*I metric and (ii) application of the sieving algorithm, which refines the results. This approach allows one to identify a plausible organization of the system in terms of non-overlapping groups of variables [24,25].

In order to analyze the hierarchical organization [4] of the system under examination, we propose an iterative version of the sieving method that groups one or more sets into a single entity to derive a hierarchy. The simplest, yet effective, way to do so consists in iteratively running the sieving algorithm on the same data, each time using a new representation in which the top-ranked relevant subset of the previous iteration, in terms of *z*I values, is considered as atomic and is substituted by a single variable (group variable). Each iteration produces therefore a new atomic group of variables: the iterations end when the *z*I of the top-ranked relevant subset falls below a preset threshold, usually equal to 3.0, that is, three standard deviations from the reference condition of variable independence [22]; see Appendix A for details.

The iterative sieving approach highlights the organization of a dynamical system by partitioning it into sets of variables detected at different iterations of the sieve. At the same time, the process of aggregation, by adding new elements to the already existing groups, allows the procedure to identify the variables of “the rest of the system” that exchange information with the subset originally under examination, discriminating in such a way between the two problematic situations of low mutual information describe above. Iteration of the “sieve and subsequent aggregation of variables” process thus allows to identify the parts of the system that can be aggregated.

Last, but not least, we note that we are not interested in analyzing all the subsets that can be drawn from the system in question, but rather we want to identify the subsets having the maximum values of the chosen index. It is therefore possible to use optimization procedures, which have the aim of finding the best values without having to go through a complete enumeration. For this purpose, we have used several heuristics in the past, including suitable variants of genetic algorithms [25,26]. Finally, it is possible to (at least partially) deal with the system’s curse of dimensionality by using parallelization strategies [27].

### 3.2. Asymptotic Null Distribution of the Empirical Integration

As anticipated, we are interested in the computation of the distribution of the empirical integration $I_{n}\left(S\right)$ when the variables indexed by *S* are stochastically independent (null distribution, or homogeneous system distribution). We will find an asymptotic (large sample) approximation that does not depend on the marginal distributions of $X_{u_{1}},\cdots X_{u_{k}}$, that is, on the specific homogeneous system chosen as a benchmark for the lack of coordination among variables.

The key observation is that the counts $n_{S}\left(x_{1},\cdots ,x_{k}\right)=n{\hat{p}}_{S}\left(x_{1},\cdots ,x_{k}\right)$ form a multinomial random vector with size *n* and class probabilities $p_{S}\left(x_{1},\cdots ,x_{k}\right)$. This gives rise to the likelihood

(11) $$\begin{array}{ccc} L_{n}\left(p_{S}\right) & \propto & \prod_{x_{1}\in X_{1}}\cdots \prod_{x_{k}\in X_{k}}p_{S}{\left(x_{1},\cdots ,x_{k}\right)}^{n_{S}\left(x_{1},\cdots ,x_{k}\right)}, \end{array}$$

where the omitted proportionality constant is the multinomial coefficient that counts the ways to group *n* observations into c(S) cells with $n_{S}\left(x_{1},\cdots ,x_{k}\right)$ observations in cell $x_{1},\cdots ,x_{k}$.

If the probabilities $p_{S}\left(x_{1},\cdots ,x_{k}\right)$ are free to vary in the standard simplex, the maximizer of (11) consists of the relative frequencies ${\hat{p}}_{S}\left(x_{1},\cdots ,x_{k}\right)$; see, for instance, Held and Sabanés Bové ([28], Ch. 5). It follows that the empirical entropy $H_{n}\left(S\right)$ equals, up to an additive constant, the negative maximized average log-likelihood $-{\bar{\ell }}_{n}\left({\hat{p}}_{S}\right)=-n^{-1}\log L_{n}\left({\hat{p}}_{S}\right)$:

(12) $$\begin{array}{ccc} {\bar{\ell }}_{n}\left(p_{S}\right) & ≐ & \sum_{x_{1}\in X_{1}}\cdots \sum_{x_{k}\in X_{k}}{\hat{p}}_{S}\left(x_{1},\cdots ,x_{k}\right)\log p_{S}\left(x_{1},\cdots ,x_{k}\right), \end{array}$$

where ≐ denotes equality up to an additive constant.

On the other hand, if the constraint $p_{S}\left(x_{1},\cdots ,x_{k}\right)=\prod_{j=1}^{k}p_{u_{j}}\left(x_{j}\right)$ is introduced, where $p_{u_{j}}\left(x_{j}\right)=P\left\{X_{u_{j}}=x_{j}\right\}$, that is, the variables indexed by *S* are assumed to be stochastically independent, the likelihood (11) can be written as

(13) $$\begin{array}{ccc} L_{n}^{0}\left(p_{u_{1}},\cdots ,p_{u_{k}}\right) & \propto & \prod_{x_{1}\in X_{1}}p_{u_{1}}{\left(x_{1}\right)}^{n_{u_{1}}\left(x_{1}\right)}\;\cdots \prod_{x_{k}\in X_{k}}p_{u_{k}}{\left(x_{k}\right)}^{n_{u_{k}}\left(x_{k}\right)}, \end{array}$$

where $n_{u_{j}}\left(x_{j}\right)$ is the count for the value $x_{j}$ in the marginal sample $X_{u_{j}}^{\left(1\right)},\cdots X_{u_{j}}^{\left(n\right)}$, for j=1,⋯,k. The maximizer of (13) clearly consists of the marginal relative frequencies ${\hat{p}}_{u_{j}}\left(x_{j}\right)=n_{u_{j}}\left(x_{j}\right)/n$, and it is apparent that $\sum_{j=1}^{k}H_{n}\left(u_{j}\right)$ equals $-{\bar{\ell }}_{n}^{\;0}\left({\hat{p}}_{u_{1}},\cdots {\hat{p}}_{u_{k}}\right)=-n^{-1}\log L_{n}^{0}\left({\hat{p}}_{u_{1}},\cdots ,{\hat{p}}_{u_{k}}\right)$ up to the same additive constant as before.

By virtue of a classical theorem of mathematical statistics, due to Wilks [29], assuming natural logarithms are used, the likelihood-ratio test statistic

$$\Lambda_{n}=2n\left({\bar{\ell }}_{n}\left({\hat{p}}_{S}\right)-{\bar{\ell }}_{n}^{\;0}\left({\hat{p}}_{u_{1}},\cdots {\hat{p}}_{u_{k}}\right)\right),$$

for large *n*, approximately follows a chi-squared distribution with degrees of freedom given by the difference in dimensions between the unconstrained and the independence statistical models, that is, equal to $d_{k}$ in (6); see ([28], Ch. 5) for a modern presentation of this result from an applied viewpoint. As $I_{n}\left(S\right)={\bar{\ell }}_{n}\left({\hat{p}}_{S}\right)-{\bar{\ell }}_{n}^{\;0}\left({\hat{p}}_{u_{1}},\cdots {\hat{p}}_{u_{k}}\right)$, the likelihood-ratio statistic can be written as $2nI_{n}\left(S\right)$ and we have an asymptotic distribution for the empirical integration. Note that, by construction, the likelihood-ratio test statistic is positive and vanishes when ${\hat{p}}_{S}\left(x_{1},\cdots ,x_{k}\right)=\prod_{j=1}^{k}{\hat{p}}_{u_{j}}\left(x_{j}\right)$, which confirms the same properties for $I_{n}\left(S\right)$.

In the special case of mutual information between two variables, where the degrees of freedom can be written as $d_{2}=\left(\right|X_{1}\left|-1)(\right|X_{2}\left|-1\right)$, the result presented above dates back to Luce [18] with a direct justification in pioneering work by Wilks [30]. If binary logarithms are used, the asymptotic chi-squared distribution applies to the statistic $2n\ln\left(2\right)\;I_{n}\left(S\right)≃1.3863n\;I_{n}\left(S\right)$; Wilks [18] uses the bit as unit of information and states the result in this form. In general, if *b* is the base used for logarithms, we can write $2n\ln\left(b\right)\;I_{n}\left(S\right)\approx \mathrm{Chisq}\left(d_{k}\right)$, where ≈ means that the left hand side has the right hand side as asymptotic distribution (approximate distribution for large *n*).

As a chi-squared distribution with $d_{k}$ degrees of freedom has mean $d_{k}$ and variance $2d_{k}$, a standardized version of the empirical integration is given by

(14) $$\begin{array}{ccc} zI_{n}\left(S\right) & = & \frac{2n\ln\left(b\right)\;I_{n}\left(S\right)-d_{k}}{\sqrt{2d_{k}}}, \end{array}$$

where b=e for nats and b=2 for bits. Note that the approximate *z*-score in (14) is consistent with the bias assessment in (5), but it also deals with sample variability; it accounts for the sample size *n* and, through $d_{k}$, for the number variables indexed by *S* and their alphabet sizes. Alternatively, one can compare subsets of different dimension using the simple normalized index $2nI/d=2nI_{n}\left(S\right)/d_{k}$, or one minus the chi-squared *p*-value of $2nI_{n}\left(S\right)$, denoted by ChSq. In Section 4, we will compare the *z*-score in (14), 2nI/d, and ChSq to the Cluster Index.

## 4. Results

In order to assess the correctness of our approach on the one hand, and to acquire new knowledge on the organization of dynamic systems on the other, we apply our relevance index methodology, or RI methodology in the following, to two very different cases. The first situation, considered in Section 4.1, involves systems in which there is no dynamic organization: each variable completes its own trajectory (in this case a random trajectory) regardless of the behavior of the other variables. By contrast, in Section 4.2, the second set of observations comes from a highly organized system, which presents two peculiar dynamic structures, capable of providing (within certain limits) self-maintenance.

### 4.1. Dynamically Homogeneous Systems

In this section, we show the results of the analysis of a set of trajectories, having different lengths, extracted from a homogeneous system composed of 21 binary variables whose two symbols have equal probability of appearing. This provides a paradigmatic example of the behavior of the RI methodology when it is applied to systems having no dynamic organization.

**Figure 1:**
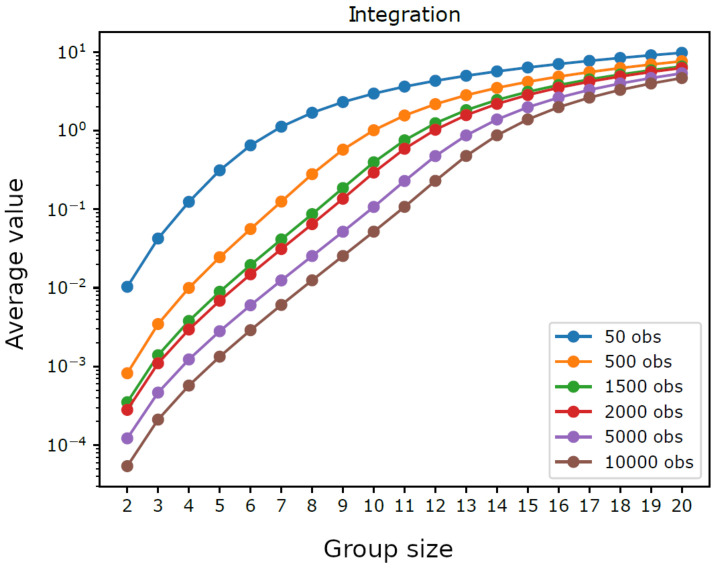
Homogeneous system. Average integration by analyzed group size (k=2,3,⋯,19,20) with varying trajectory length (*n* = 50, 500, 1500, 2000, 5000, and 10,000).

**Figure 2:**
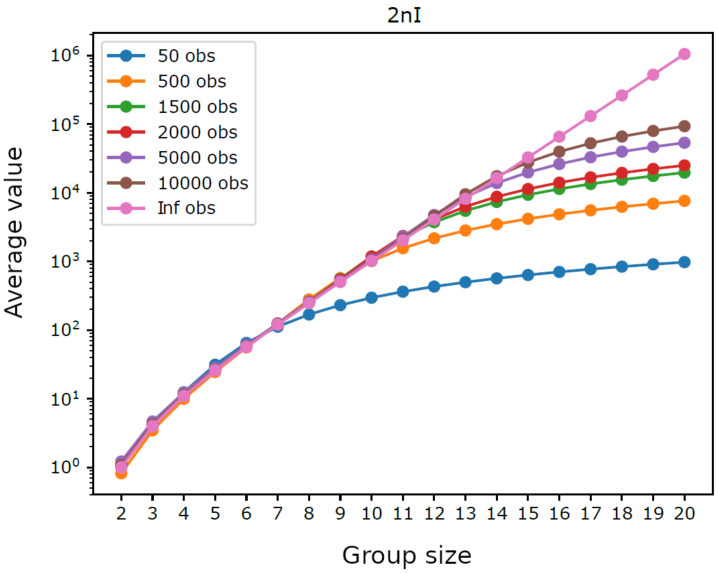
Homogeneous system. Average of $2nI=2nI_{n}\left(S\right)$, by group size k=|S|, for six different trajectory lengths, compared with the mean $d=d_{k}$ of the chi-squared distribution (average with infinite observations).

**Figure 3:**
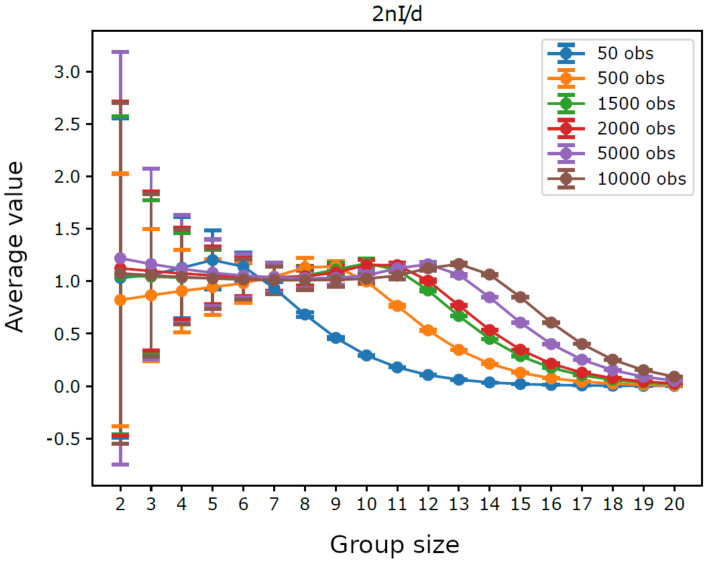
Homogeneous system. Average of $2nI/d=2nI_{n}\left(S\right)/d_{k}$, by group size k=|S|, for six different trajectory lengths, with error bars at three times the standard deviation of observations.

Figure 1 displays the average integration by group size for trajectories having different lengths. For intermediate group sizes it was not feasible to consider all possible groups: if the number of groups with a given size exceeded the threshold 10,000, we performed a sampling and used only 10,000 randomly extracted groups (for each analyzed size). We can observe that shorter trajectories, as well as larger groups, lead to higher average integration, which reflects the bias term $d_{k}/2n$ in (5). Indeed, as illustrated in Figure 2, the average values of $2nI_{n}\left(S\right)$ do not depend on *n* for small group sizes, which shows that in such cases the chosen trajectory lengths are sufficient to provide a reliable estimate of integration. Furthermore, it can be seen in Figure 3 that the average values of $2nI_{n}\left(S\right)/d_{k}$ are close to 1.0 for small groups sizes, which confirms the reliability of such estimates. Note that the maximum group size, such that the estimated integration is reliable, grows with the number of observations, from k=7 with n=50 observations to k=14 with *n* = 10,000 observations, which is consistent with the fact that $d_{k}$ can be seen as the average of $2nI_{n}\left(S\right)$ with infinite observations. Note as well that the width of the error bars in Figure 3 is greater for smaller group sizes, where there are few distinct groups with little overlap between them, while it drops monotonically with the increasing length of the trajectories.

Figure 3 shows that the average of $2nI_{n}\left(S\right)/d_{k}$ quickly drops below the value 1.0 for large groups. This fact indicates that, in the trajectories examined, the values of $2nI_{n}\left(S\right)/d_{k}$ for these groups are small compared to what they should be in order to thoroughly observe large groups: large size groups are not very evident, or, in other words, they are difficult to detect. Indeed, the same limit holds for groups formed by a few variables having a large number of symbols. Actually, systems with a number of degrees of freedom greater than the number of observations necessarily cannot be fully observed, which results in a fundamental limit regarding the reliability of their identification. As as special case, this limit holds also for the group variables formed by the aggregation of simpler variables happening during to the iteration of the sieving algorithm, a process which creates new group variables with a number of symbols adequate to maintain all the information carried by the original ones. In such a way, groups formed by few variables with a large number of internal levels maintain the same number of degrees of freedom as groups formed by a large number of simpler variables, proposing again the critical situation. To sum up, however, this limitation depends on the number of performed observations rather than on the method used for the detection of groups.

The above-described limit puts a natural end to the process of iterative grouping described in Section 3.1. However, even before that limit is reached, the process should be stopped if larger interacting groups are not present. This will be done on the basis of a method-specific assessment of the probability of making a mistake during the progress of the agglomeration process. To this aim, in the following, we study the performance of our proposed relevance index, the approximate *z*-score of integration (*z*I) given by (14), in comparison with three other relevance indices, which can replace it in the sieve: the *z*-score of Tononi’s Cluster Index (*z*CI), the simple normalized index $2nI/d=2nI_{n}\left(S\right)/d_{k}$, and one minus the chi-squared *p*-value of $2nI_{n}\left(S\right)$, denoted by ChSq.

**Figure 4:**
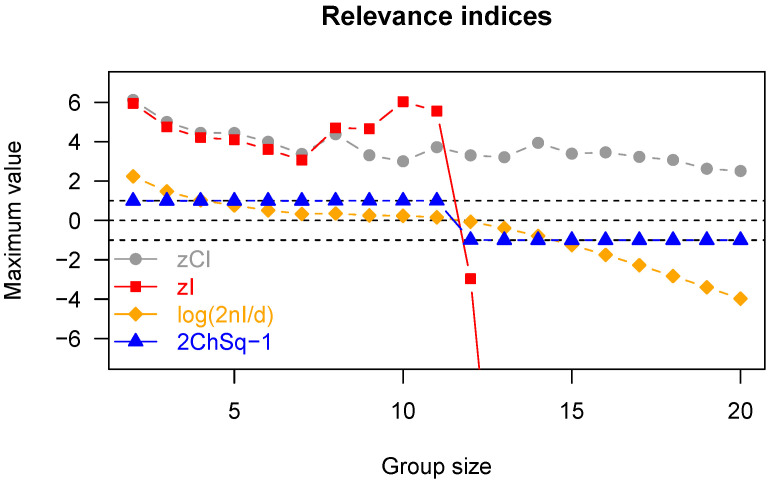
Homogeneous system. Maximum values of the four relevance indices under comparison (*z*CI, *z*I, 2*n*I/*d*, ChSq). The indices 2*n*I/*d* and ChSq are transformed so that they have zero as the null value (like *z*CI and *z*I). The horizontal dashed lines mark the lower and upper bounds for ChSq, and the null level. The dramatic drop in the indices based on the chi-squared distribution after dimension 12 indicates that the approximation used (that of assuming infinite observations) is no longer valid. Such knowledge is not available in the case of the *z*CI index, which we calculated in each dimension resorting to a very onerous bootstraps procedure.

Figure 4 shows the maximum values of the four indices for each group size, sorted by group size. Basically, in a homogeneous system, there should not be any relevant subset, although some will spuriously show up due to the finiteness of the number of observations. A high threshold on the relevance index should be able to limit the frequency of such spurious appearances. In this respect, the plot in Figure 4 suggests that ChSq is unfit to the task, because it gets very close to its upper bound for all interesting group sizes. Furthermore, it can be seen from Figure 4 that the variability of 2nI/d decreases with group size, which makes the emergence of smaller spurious groups more frequent (for a given threshold); this is clearly an undesirable feature. On the other hand, it appears that *z*CI and *z*I have comparable maxima across interesting group sizes; recall, however, that the computation of *z*CI is onerous, whereas that of *z*I is not, which makes *z*I preferable to *z*CI in practice.

**Figure 5:**
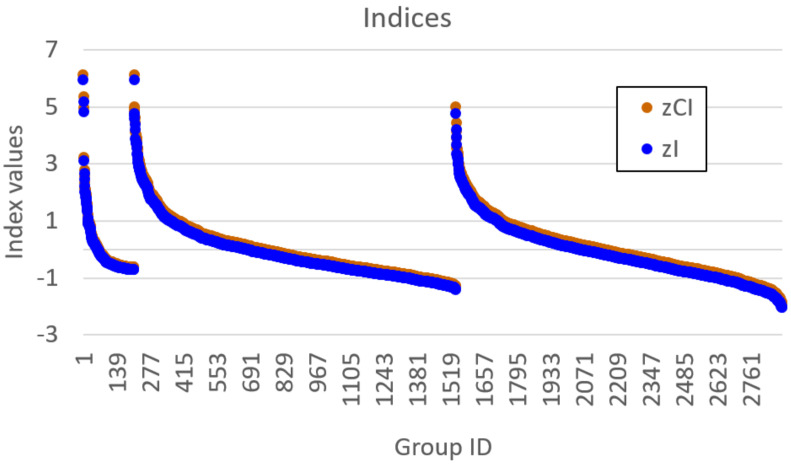
Homogeneous system. Values of *z*CI and *z*I for each single group of up to four variables, sorted by group size and then by *z*I.

Figure 5 focuses on the distribution of *z*CI and *z*I in the smallest groups. It can be seen that each group size presents several extreme values, which indicates that several groups could spuriously exhibit some coordination activity. Remarkably, within each group size, the two indices provide the same ranking (convey the same information). We shall see in the next section that this is not necessarily the case for non-homogeneous systems.

### 4.2. Dynamically Organized Systems

In the previous section, we used data from a homogeneous system to verify the effectiveness of the chi-squared distribution and to make some general remarks on the application of our relevance index to the evaluation of the degree of dynamic organization of subgroups of variables. Our goal, however, is to look for groups that are dynamically relevant (relevant subsets), whose identification can facilitate the understanding of the system’s dynamic organization. To this aim, the homogeneous system plays the role of a yardstick, while we are interested in analyzing dynamically non-homogeneous systems.

There are two different ways of searching for relationships present within a dynamical system. The first strategy consists in juxtaposing several separate instances of the same organization; the other strategy is that of observing one particular trajectory, possibly disturbing it from time to time. Indeed, as we are adopting entropic measures, we do not actually make use of the hypothesis of having all states successor of one another: changing the order of the observations does not influence the frequency of each state. We can therefore use the same framework for both situations. Indeed, we analyzed several systems with strong dynamical organization, by juxtaposing the states belonging to different asymptotic behaviors of the same system (different attractors of a genetic regulatory network [20,31,32] and patients affected by the same kind of disease [33]) or by observing the trajectory of a single system (a socio-economic system [34]), sometimes perturbing it (metabolic networks [24] and autocatalytic systems [19,35]). The performed RI analyses show some common characteristics, so in this paper we choose to expose them by commenting in detail a particular system: an autocatalytic reaction network introduced in [35].

The situation concerns the formation of groups of molecules able to collectively catalyze and self-replicate, a process that is thought to be fundamental for the origin of life [36,37,38,39,40,41,42] and is likely to play an important role also in future biotechnological systems [43]. Indeed, currently living beings are based on self-replicating chemical structures, where the presence of enzymes (biological catalyzers) plays an essential role. A useful representation of such systems is based on Reflexive Autocatalytic Food (RAF)-generated sets [44,45], a sophisticated description recently utilized in biochemical contexts [44,46,47,48] or in protocell architectures [49] to characterize structures with different kind of interactions (production and catalysis).

**Figure 6:**
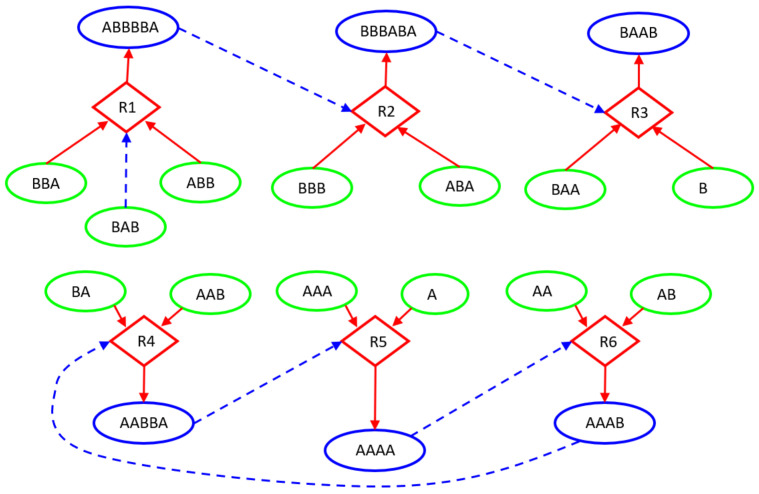
CSTR26 system. The chemical system under analysis. Circular nodes depict chemical species: the green ones stand for those injected into the Continuous-flow Stirred-Tank Reactor (CSTR) (food species) and the blue ones represent the more complex species built by specific concatenations of the food species. Diamond shapes represent reactions, where incoming arrows go from substrates to reactions and outgoing arrows go from reactions to products. Dashed lines indicate the catalytic role of a particular molecular species within the specific reaction context. For instance, thanks to the catalyst BAB, reaction R1 combines the food species ABB and BBA into the complex ABBBBA, while reaction R3 combines the food species BAA and B into the complex BAAB, when the catalyst BBBABA is present.

**Figure 7:**
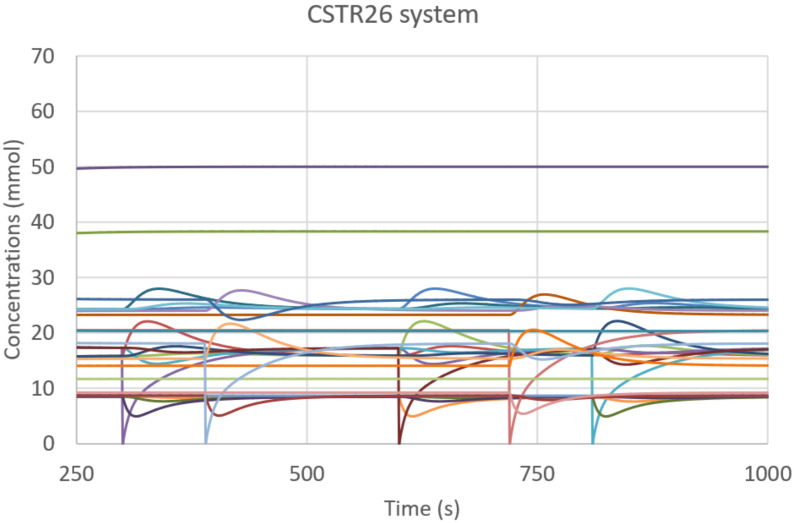
CSTR26 system. The system behavior within the time interval under analysis, including the externally generated perturbations in order to stimulate the dynamic response of the system. The kinetic constants of all present reactions have the same value *k* = 0.0025 s^−1^ mol^−1^; the incoming concentration of each food species is 0.001 M, while each second 2% of the CSTR volume is renewed.

In this paper, we deterministically simulate two particular instances of RAFs: a linear chain of reactions, having the root with existence guaranteed from the outside, and a ring, in which the substances produced are catalysts for at least one of the other substance to be produced. The two structures are immersed in a Continuous-flow Stirred-Tank Reactor (CSTR) [50] featuring a constant influx of feed molecules, constantly present in the incoming flow of the CSTR and therefore playing the role of the “food” species at the base of RAF arrangements, and a continuous outgoing flux of all the molecular species proportional to their concentration. The simulations are based on a relatively simple system inspired by a model used in [51,52,53] and originally proposed by Kauffman [41,54]. The scheme simulated in this paper, represented in Figure 6, involves only enzymatic condensations, decomposed in three steps: the first two steps create (and destroy in an overall reversible process) a temporary complex, composed by one of the two substrates (the “first substrate”) and the catalyst, which is combined in the third step with the other substrate to release the catalyst and the final product; see in [49] for a more accurate description. The dynamics of the systems are simulated adopting a deterministic approach: the reaction scheme is translated into a set of ordinary differential equations ruled by the mass action law [49,55] and integrated by means of an Euler method with step-size control. Figure 6 represents the simulated system and Figure 7 illustrates its dynamic behavior.

The asymptotic behavior of this kind of system is a single fixed point [56], which does not provide any useful observation for identifying the underlining dynamic structure; in order to apply our analysis, indeed we need to observe the feedbacks in action. We then follow a perturbative approach, consisting in disturbing the asymptotic behavior and recording the consequent transient: we temporarily lower, one by one, by two orders of magnitude, the input concentrations of the food species (green ellipses in Figure 6) after the system has reached its stationary state. In order to analyze the system response to perturbations, we use a three-level coding where, for each species, the digits 0, 1, and 2 stand for “concentration decreasing”, “no change”, and “concentration increasing”, respectively. Specifically, in this experiment, we consider the concentration of a chemical species as being constant if it has not changed by more than 1% in a given time period (ten seconds in the example of Figure 7).

**Figure 8:**
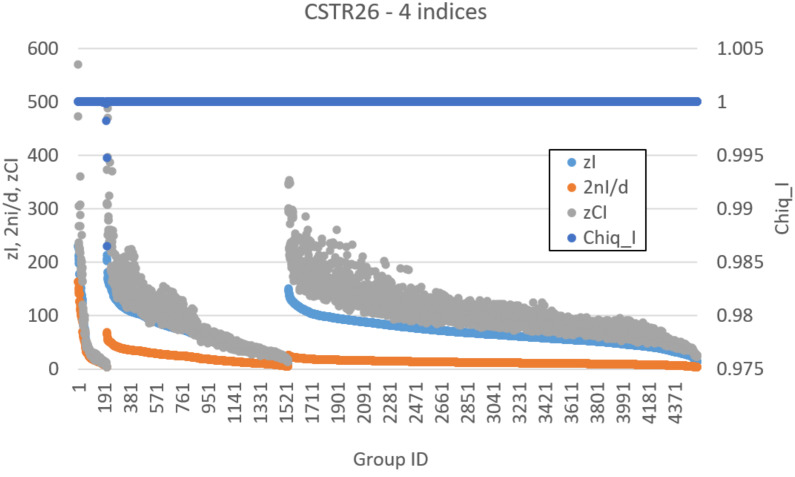
CSTR26 system. Values of *z*CI, *z*I, 2*n*I/*d*, and ChSq (right vertical axis) for each single group of up to four variables, sorted by group size and then by *z*I.

Let us now analyze the findings of our algorithm. Figure 8 represents the distribution of *z*I, *z*CI, 2nI/d, and ChSq in the smallest groups of variables. The indices 2nI/d and ChSq show no discriminatory power. The indices *z*I and *z*CI no longer provide the same information, as it happened in the homogeneous case. In particular, a detailed group by group analysis has shown that the presence of numerous dynamically organized assemblies that interact with each other does not allow the *z*CI index to correctly identify the relevant parts. As an example of such interactions, in Figure 6, the couple of variables BBB and ABA is in strong association (with different intensities) with the variation of concentrations of ABBBBA and BBBABA, or with the variation of concentrations of BAA or BAAB. In the present case, as previously commented, low values of mutual information can derive from a low information exchange between the subgroup and every element of the rest of the system or from a high exchange of information between the subgroup and a small part of the rest of the system, while the other parts are not involved in the exchange. The two situations have to be discriminated, but the mutual information is not providing the correct information to satisfy the need. On the other hand, if we consider the iterated sieve guided by the values of *z*I, we observe that the process of growing already existing strong groups makes it possible to gradually identify the variables to be aggregated, avoiding the difficulty and discriminating in such a way the situations otherwise giving almost similar and low mutual information values. For these reasons, the index *z*I appears to be preferable to the index *z*CI.

**Figure 9:**
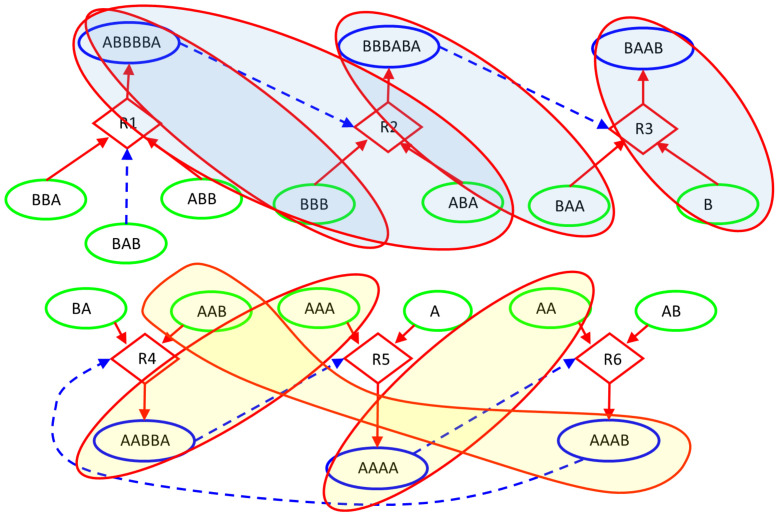
CSTR26 system. The first groups identified by our RI analysis: catalyst-first substrate pairs, and the terminal pair of chemicals within the linear chain of reactions.

**Figure 10:**
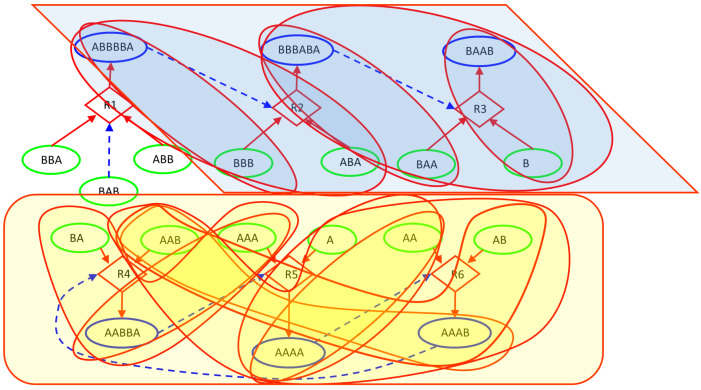
CSTR26 system. The final situation identified by our RI analysis, in which the action of the iterated sieve induces the formation of a hierarchy of encapsulated groups.

Figure 9 and Figure 10 illustrate the analysis of the CSTR system using the *z*I index. Remarkably, the final groups (the relevant sets object of the research) correctly identify the two dynamic organizations we have included in the system (Figure 10). The three variables—BBA, BAB, and ABB—were not significantly involved in any perturbation event, and they are correctly outside any group. The presence of subgroups within the relevant sets indicates the presence of a hierarchy: the identified dynamic organizations are composed of smaller parts. In the case of an unknown system, the search for relations between the parts will be investigated by experts in the field. In our case the ground truth is known, and we can appreciate the order in which the algorithm evaluates the coordination evidences. First, as shown in Figure 9, the analysis merged the catalyst–“first substrate” pairs. Recall that, in the system under examination, the catalytic action is carried out through the formation of a short-lived active complex, composed by the catalyst and one of the substrates (the “first substrate”). If later on the complex meets the other substrate (the “second substrate”), the reaction proceeds releasing the catalyst and producing the final product; otherwise, the complex dissociates releasing the two species of which it is composed.) (actually having very strong relationships within the system) and the terminal pair of the linear chain (each perturbation of a chemical species belonging to this system actually producing a coordinated signal in this pair). After that, the “second substrate” was added to each group with catalyst, and finally further mergers were made, until the analysis reached the threshold for *z*I and stopped in the situation of Figure 10.

## 5. Conclusions

A formidable challenge in Complexity Science is that of identifying emergent organizations in complex dynamical systems, a theme with broad applications. A central question is then that of detecting the emerging structures: these may be either static entities or dynamical patterns, or some mixture of the two. Identifying these configurations is seldom simple, because of the more-than-binary relationships among variables, the possible multiple memberships of system entities, or the fuzziness of boundaries among groups. A large part of the current approaches aiming at the detection of these objects is based on network representations and static features, such as link distributions or topologies, whereas the system’s elements may work in a coordinated manner even though they are not directly linked, because of the dynamical laws governing the system.

In this paper, we presented a methodology for the identification of mesolevel objects, which we call relevant subsets, based on entropic measures, which may involve dynamical aspects [19,24,27,34] or juxtapose different realizations within a population of individuals sharing the same common organization [20,31,33]. We identified an entropic measure useful for the detection of relevant subsets and studied its theoretical distribution, a fact that helps in the interpretation of the results and allows to avoid the excessively onerous bootstrap calculations from a homogeneous system that are needed to compare groups of different size. Finally, we showed that the increase in the number of observations allows the identification of larger and larger groups, up to the asymptotic case of complete observability in the case of infinite observations. As an example of application, we analyzed a paradigmatic case regarding the identification of autocatalytic sets of reactions, a chemical situation related to the origin of life problem.

The general schema for the identification of relevant subsets presented in this paper is a promising approach for a difficult task: we showed some interesting results, while remarking that there are still open questions to answer. A delicate aspect concerns the number of observations necessary to identify large groups, which makes it useful to search for statistical corrections in case of few of them. In this regard, more elaborate entropy estimators [13,14,15] could be helpful. Furthermore, it should be observed that the current version of our method is based on the classical measure introduced in information theory by Claude Shannon (and sometimes referred to as the Boltzmann–Gibbs–Shannon entropy). It is well known that different definitions of entropy have been proposed including those of Tsallis and Renyi, which have interesting features, while lacking the additivity of Shannon’s; see, e.g., in [57] for a recent review. We think that a generalization of the RI method based on these nonadditive entropies might lead to interesting results, in particular in complex systems with long-range interactions. Finally, it can be observed that the iterated sieving algorithm presupposes an at least partial decomposability of the dynamical organization into separate parts. On the other hand, the iterated and progressive recomposition decreases, at each iteration, the number of degrees of freedom of the model representing the system, and if correct it could allow to identify more easily (or with fewer observations) large groups. We think that these questions deserve further investigation in future works.

## Figures and Tables

**Figure A1:**
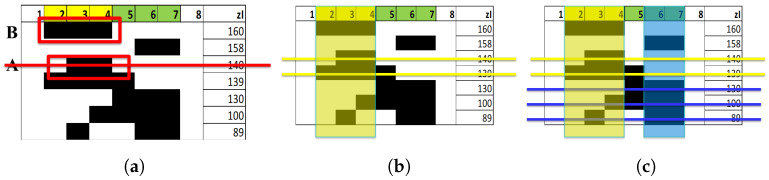
Sieving method. (**a**) If A is a subset (or a superset) of B, and B ranks higher than A, then we can think that A is an epiphenomenon, and B a more fundamental element than A (only the first part of the list of all possible groups is shown in the figure). (**b**) The sieving algorithm, applied to the strongest set, eliminates some groups (yellow lines). (**c**) The procedure is then repeated starting from the strongest remaining set and iterated until there are no more subsets or supersets to be eliminated. In the figure, the two remaining sets of the visible part of the list are those corresponding to index values 160 and 158.

**Figure A2:**
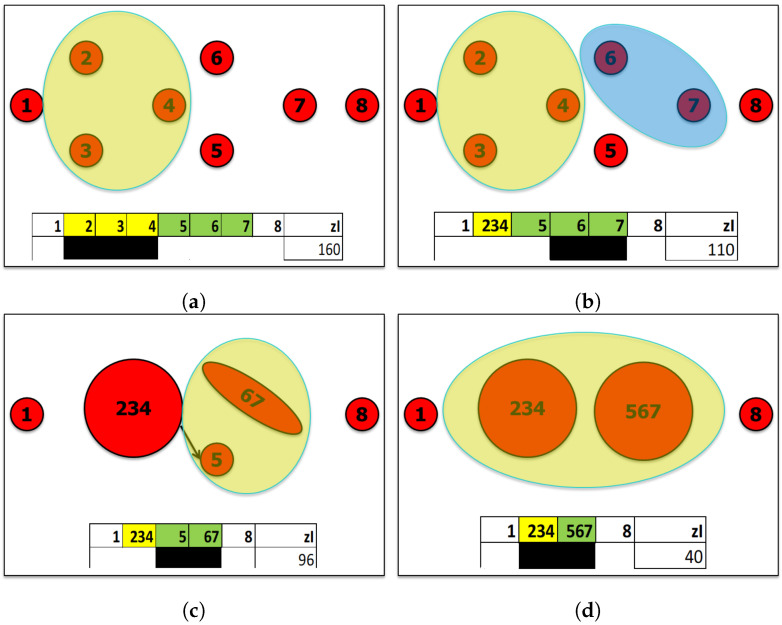
Iterative sieving method. (**a**) The first iteration of the sieving algorithm identifies the three variables 2, 3, and 4 as the top-ranked building block and compacts them into a single group variable. The new variable has a number of levels equal to or greater than the maximum number of levels of its components, but typically (much) less than their Cartesian product, because the variables are dynamically dependent. The new variable is inserted in the observations in place of the three from which it is composed. (**b**) The subsequent analysis identifies the group composed of the variables 6 and 7, which will then be compacted and replaced by the group variable {6,7}. (**c**) The variables {6,7} and 5 are further merged into the group variable {5,6,7}. (**d**) The variables {2,3,4} and {5,6,7} are merged into the variable {2,3,4,5,6,7}. The algorithm will not accept further mergers, because the groups {1,2,3,4,5,6,7}, {2,3,4,5,6,7,8}, and {1,2,3,4,5,6,7,8} have a *z*I lower than the chosen threshold (situation not shown). Note that only one group is compacted at a time, and so it is possible to avoid calculating the eliminations due to the simple sieving algorithm; see also the final paragraph of this Appendix A.

**Figure A3:**
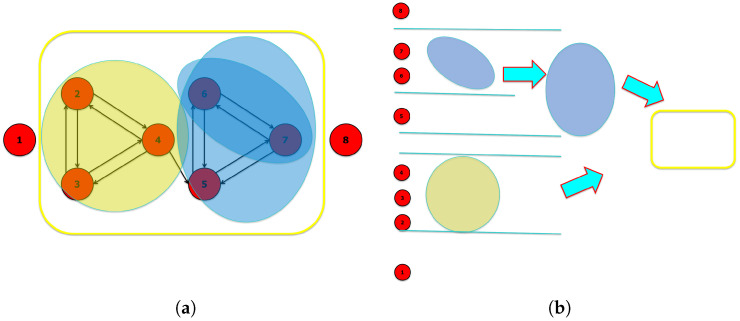
Iterative sieving method. (**a**) The nesting of the observed dynamic groups (superimposed on the system variables). The variables 1 and 8 are in fact random variables separated from the system at work (the largest group identified, highlighted here by the yellow box). Each variable performs the XOR of its input variables, except for node 5 that sets node 4 in AND with the XOR of nodes 6 and 7. (**b**) The hierarchy identified by the succession of groupings carried out by the *z*I analysis.

## Data Availability

The data analyzed in this study are publicly available and can be found here: http://morespace.unimore.it/marcovillani/software/, accessed on 26 March 2021.

## Appendix A

### Appendix A.1. The Sieving Method

The collection of sets returned by our proposed analysis is likely to contain groups included in others or partially overlapping groups, a fact that requires further analyses to assess their actual relevance. Having a high index value is not sufficient to characterize a set, because such ranking might result from the inclusion of a smaller set of variables characterized by an even higher *z*I, i.e., the set under consideration could be a superset of a more relevant one. In this case, the only relevant set would be the latter. On the other hand, a set having a high *z*I value might reach an even higher value, if some other relevant variables are added to it, i.e., the set under consideration could be a subset of a more relevant one. In this case, we would consider only the larger set as relevant.

In order to tackle this problem, we proposed a postprocessing sieving algorithm to reduce the overall number of subsets. The main assumption of the procedure is that if *A* is a proper subset of set *B*, that is, A⊂B, then only the higher value subset is taken into consideration, see Figure A1 [22]. Therefore, only disjoint or partially overlapping subsets are kept by the sieving algorithm.

After the sieve has been applied, the remaining subsets cannot be decomposed any further, and thus they represent the building blocks of the dynamical organization of the system [24,25]. We refer to these building blocks as Candidate-Relevant Sets (CRS) in the following pseudocode of the sieving method (Algorithm A1).

**Algorithm A1** The Sieving Method
**Input:** The array *S* of all the subsets, ranked by their index value in descending order **Output:** CRS, a subset of *S* CRS ⇐ ∅ *n* ⇐ |S| Initialize auxliary array Aux[*k*] ⇐ 0 for *k* in 1...*n* **for***i* = 1 to *n* − 1 **do** **for** *j* = *i* + 1 to n **do** **if** Aux[*i*] ≠ 1 **and** Aux[*j*] ≠ 1 **then** **if** *S*[*i*] ⊂ *S*[*j*] **or** *S*[*j*] ⊂ *S*[*i*] **then** Aux[*j*]⇐ 1 **for***i* = 1 to *n* **do** **if** Aux[*i*] = 0 **then** CRS ⇐ CRS ∪ {*S*[*i*]}

### Appendix A.2. The Iterative Sieving Method

The method previously described allows one to identify a plausible organization of the system in terms of its lowest level, possibly overlapping, subsets of variables. Nevertheless, as complex systems have often a hierarchical structure, one may want to be able to make hypotheses on aggregated relations among the dynamic building blocks thus identified. To this aim, we devised an iterative version of the sieving method, which acts on the data by iteratively grouping the variables in one or more building blocks into a single entity. There are several ways to do so, but the simplest one, yet quite effective, consists in iteratively running the sieving algorithm on the same data, each time using a new representation in which the top-ranked building block of the previous iteration is considered as atomic and substituted by a single variable (henceforth called a group variable). In this way, each run produces a new atomic group of variables composed of both single variables and group variables introduced in previous iterations [22].

Let us consider a synthetic example—of which we therefore know the connection topology. The system includes eight variables, denoted by 1,⋯,8, and suppose that the group {2,3,4} is the most relevant set detected by the first iteration of the algorithm. The second iteration will then analyze the dynamics of a system comprising the six variables 1, {2,3,4}, 5, 6, 7, and 8; the third iteration will analyze, for instance, the dynamics of a system comprising the five variables 1, {2,3,4}, 5, {6,7}, and 8; and so on until the index value of the most relevant set detected falls below a predetermined threshold, which we usually set equal to 3.0, see Figure A2.

The succession of mergers performed by the iterated sieving algorithm allows to observe a hierarchy of nested groups: the final groups are the largest possible groupings, which constitute our Relevant Sets, see Figure A3.

The above-described version of the iterative sieve is quite effective. As only one group is compacted at a time, there is no need to calculate the eliminations due to the simple sieve. Nevertheless, other variants may be implemented, which may produce more than one group variable per iteration: instead of considering just the top-ranked building block as a new group variable, one may possibly transform the first *q* sets into group variables, with *q* chosen according to some empirical criterion.
